# Inter-epidemic abundance and distribution of potential mosquito vectors for Rift Valley fever virus in Ngorongoro district, Tanzania

**DOI:** 10.3402/gha.v8.25929

**Published:** 2015-01-21

**Authors:** Clement N. Mweya, Sharadhuli I. Kimera, Lesakit S. B. Mellau, Leonard E. G. Mboera

**Affiliations:** 1 Tukuyu Research Centre, National Institute for Medical Research, Tukuyu, Tanzania; 2 Department of Veterinary Medicine and Public Health, Sokoine University of Agriculture, Morogoro, Tanzania; 3 National Institute for Medical Research, Dar es Salaam, Tanzania

**Keywords:** Rift Valley fever, mosquitoes, abundance, distribution, inter-epidemic period, Tanzania

## Abstract

**Background:**

Rift Valley fever (RVF) is a mosquito-borne viral zoonosis that primarily affects ruminants but also has the capacity to infect humans.

**Objective:**

To determine the abundance and distribution of mosquito vectors in relation to their potential role in the virus transmission and maintenance in disease epidemic areas of Ngorongoro district in northern Tanzania.

**Methods:**

A cross-sectional entomological investigation was carried out before the suspected RVF outbreak in October 2012. Mosquitoes were sampled both outdoors and indoors using the Centre for Disease Control (CDC) light traps and Mosquito Magnets baited with attractants. Outdoor traps were placed in proximity with breeding sites and under canopy in banana plantations close to the sleeping places of animals.

**Results:**

A total of 1,823 mosquitoes were collected, of which 87% (*N*=1,588) were *Culex pipiens* complex, 12% (*N*=226) *Aedes aegypti*, and 0.5% (*N*=9) *Anopheles* species. About two-thirds (67%; *N*=1,095) of *C. pipiens* complex and nearly 100% (*N*=225) of *A. aegypti* were trapped outdoors using Mosquito Magnets. All *Anopheles* species were trapped indoors using CDC light traps. There were variations in abundance of *C. pipiens* complex and *A. aegypti* among different ecological and vegetation habitats. Over three quarters (78%) of *C. pipiens* complex and most (85%) of the *A. aegypti* were trapped in banana and maize farms. Both *C. pipiens* complex and *A. aegypti* were more abundant in proximity with cattle and in semi-arid thorn bushes and lower Afro-montane. The highest number of mosquitoes was recorded in villages that were most affected during the RVF epidemic of 2007. Of the tested 150 pools of *C. pipiens* complex and 45 pools of *A. aegypti*, none was infected with RVF virus.

**Conclusions:**

These results provide insights into unique habitat characterisation relating to mosquito abundances and distribution in RVF epidemic-prone areas of Ngorongoro district in northern Tanzania.

Rift Valley fever (RVF) is a mosquito-borne viral infection of both ruminants and humans (1, 2). The disease occurs as intermittent epidemics with intervals of 10–15 years, mainly after periods of exceptionally heavy rainfall (3). In Tanzania, major outbreaks were reported in Ngorongoro district in 1997–1998 and 2006–2007 causing massive abortions and deaths in livestock. The 2006–2007 outbreak was reported in 25 districts in Arusha, Manyara, Kilimanjaro, Tanga, Dodoma, Iringa, and Morogoro regions of the country causing 144 human deaths (4, 5). Previous studies in other RVF outbreak areas indicated that RVF epidemics are associated with the distribution and increased population of mosquito vectors (6–10) .

Several mosquito species are able to act as vectors for transmission of the RVF virus (11–17) . However, the dominant vector species varies between different regions, and different species can play different roles in sustaining the transmission of the virus. Studies of mosquito vectors in north-eastern Kenya have shown that *Anopheles squamosus*, *Aedes ochraceus*, and *Aedes mcintoshi* play major roles as local important vectors (10–12, 18). Many mosquito species have demonstrated the capability to transmit the virus to animals (6, 7, 19, 20). *Culex pipiens* complex and *Aedes aegypti* are the major RVF mosquito vectors located in many disease endemic areas (21–27) . The RVF virus is spread primarily by the bite of infected mosquitoes, mainly the *C. pipiens* complex and *Aedes* species, which can acquire the virus from feeding on infected animals. The *Aedes* female mosquito is also capable of transmitting the virus trans-ovarially to her offspring via eggs leading to new generations of infected mosquitoes hatching from eggs. The RVF virus persists trans-ovarially within mosquito eggs that can survive for several years in dry conditions (18).

Attempts to implement early-warning systems and effective surveillance strategies for epidemics require an understanding of the abundance and distribution of mosquito vectors with transmission patterns of the disease. However, this has often been hindered by a lack of reliable information on mosquito vectors responsible for the occurrence and persistence of the disease during epidemic and inter-epidemic periods (IEP). In Tanzania, like in many parts of Africa, little is known about RVF mosquito vectors abundance and transmission intensities (7). It was the objective of this study to determine the abundance and distribution of mosquito vectors in relation to their potential role in the RVF virus transmission and maintenance in disease epidemic areas of Ngorongoro district in northern Tanzania.

## Materials and methods

### Study area

**Fig. 1:**
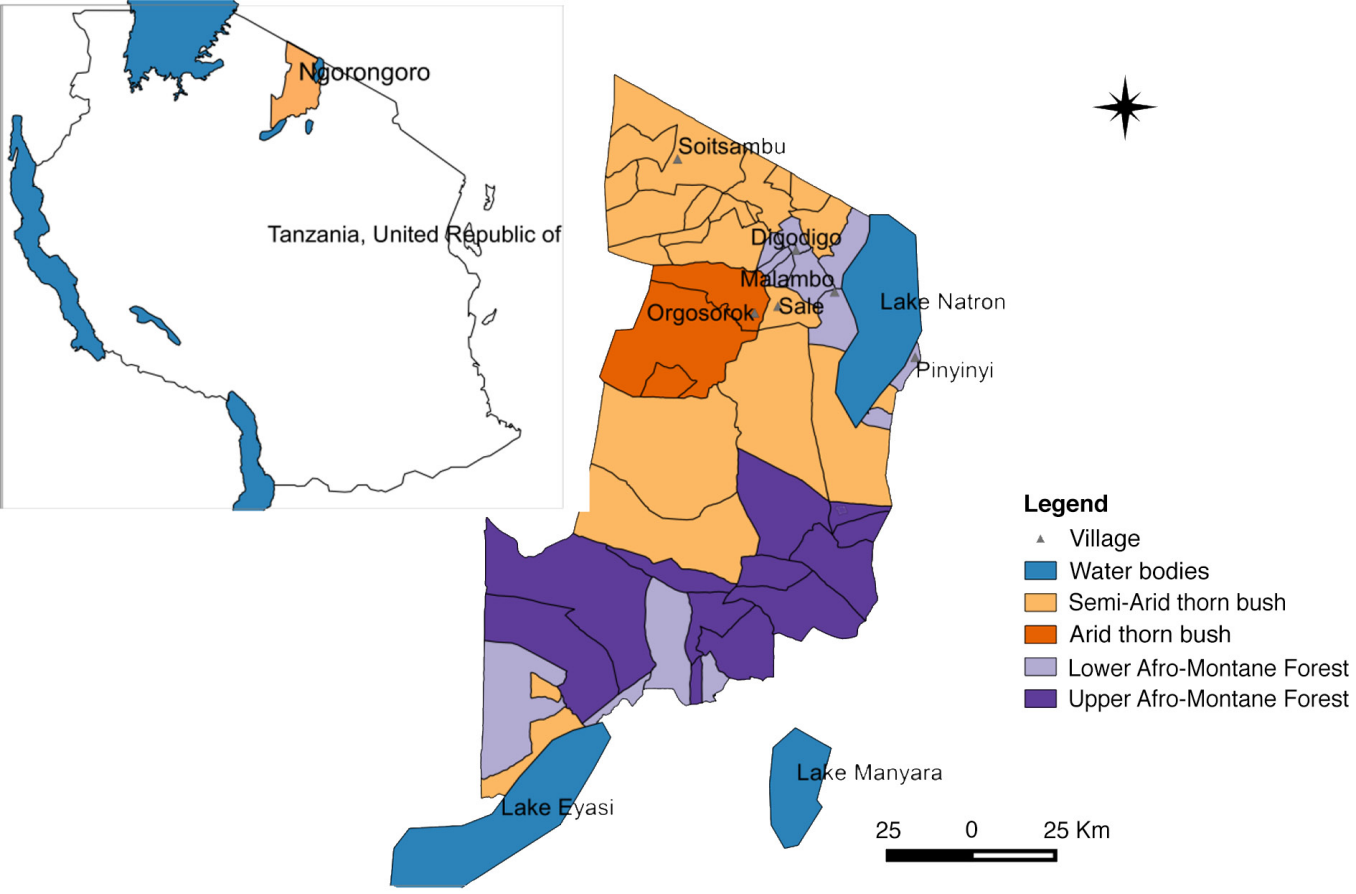
Map of Ngorongoro district indicating sites where mosquito collections were done and habitat characteristics such as vegetation features.

**Fig. 2:**
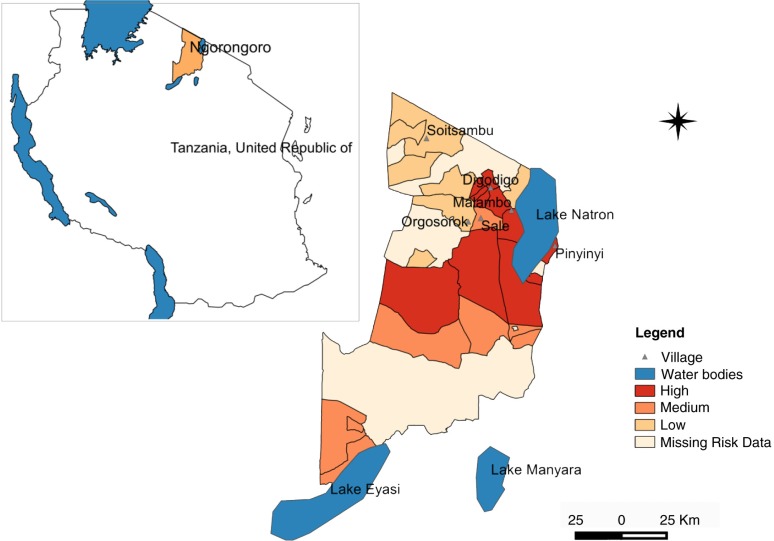
Map of Ngorongoro district indicating level of impact due to RVF epidemic in 2006–2007 as distributed in three levels of risk, high, medium, and low.

This study was carried out in Ngorongoro district (2°S45′50.4″, 35°E34′04.8″) in northern Tanzania (Figs. 1 and 2). The district is within the Serengeti–Masai Mara ecosystem defined by the limits of the annual wildlife migration. It represents a unique interaction between livestock, wildlife, and humans while involving animal migration from neighbouring Kenya, which has experienced similar RVF outbreaks since the 1930s. The district has been described as the main RVF hotspot area during 2006–2007 (5).

The study area falls under a semi-arid rangeland area in the Rift Valley having bimodal rainfall with a long rainy season in March–May and a short rainy season (October–December). The total amount of annual rainfall ranges from 700 to 1,800 mm with a mean monthly temperature of 19°C. The district has extreme habitat diversity comprising areas of subalpine pasture at 4,000 m, montane evergreen forest, arid thorn bush, and treeless short grass plains at 1,800 m. The vegetation mainly consists of various shrubs and acacia bushes. Livestock species kept are primarily cattle, goats, sheep, and donkeys. This study involved mosquito collection in six villages namely Orgosorok, Soitsambu, Digodigo, Malambo, Sale, and Pinyinyi.

### Mosquito collection

Mosquito collection was carried out in the dry season from September to October 2012 before the suspected RVF outbreak (28). All outbreaks in East Africa have been reported to occur following periods of abnormal drought, followed by abnormal heavy rains and the consequent emergence of large numbers of *Aedes* and *Culex* mosquitoes (18, 29–31) . Three Mosquito Magnets and three Centre for Disease Control (CDC) light traps were set for three consecutive days both outdoors and indoors. Outdoor mosquito collections were made using either unbaited or Octenol-baited Mosquito Magnets (Cordless LibertyPlus) and Carbon dioxide-baited CDC light traps (John W. Hock Company, Gainesville, FL, USA). Indoor mosquito collections were made using unbaited CDC light traps. Traps were set using the procedure previously described in other studies 32–34) . Outdoor traps were placed in proximity with potential breeding sites and under canopy in banana plantations and in proximity to animal shelters.

Habitat characteristics data such as topography and vegetation types were recorded and observed during the study period for each village surveyed.

### Mosquito identification

Using a microscope, mosquitoes were identified to genus or species level using morphological keys (35, 36). Due to intraspecific variability of morphometrical characteristics, inconsistencies in original descriptions of morphological characters, the small size of the structures being observed under the light microscope that would require identification using molecular techniques, all *Culex* species were grouped together as *C. pipiens* complex to possibly include *C. pipiens pipiens*, *C. pipiens quinquefasciatus*, *C. pipiens torrentium*, *C. pipiens molestus*, and so on. Mosquitoes that could not be identified morphologically directly in the field due to minor specimen damage such as a missing leg or wing were sent to the Amani Research Centre of the National Institute for Medical Research (NIMR) for further identification assistance. Mosquitoes were then killed and kept on ice during transportation to the laboratory.

### Molecular detection of RVF virus

Collected mosquitoes were kept on ice initially and then transported to the laboratory for RNA extraction. Transportation of mosquito samples from field to laboratory was done after every 7–12 days. Pools of 5–10 mosquitoes belonging to the same species were ground before RNA was extracted using TRIzol^®^ viral RNA/DNA kit (Invitrogen, Corp), following manufacturer's instructions. Extracted RNA kept in RNase-free water and stored at −20°C was used in real-time reverse transcription polymerase chain reaction (qRT-PCR) for RVF virus detection. The LightMix qRT-PCR kits for RVF virus and Light Cycler FastStart DNA Master HybProbe reaction mix (Roche) was used for the qRT-PCR with the Light Cycler 2.0 thermocycler (Roche) for all samples (37, 38).

### Data analysis

Data was entered and summarised in Microsoft Excel spread sheets. Mosquito abundance was calculated as the number of mosquitoes collected in each village. Mosquito abundance was compared with area, which had a high RVF risk level for the 2006–2007 epidemics. The Chi-square test was used to test significance of the differences in abundance according to the characteristics of the mosquito collection site, vegetation type in proximity, and topographical characteristics of the village with a risk level according to the 2006–2007 epidemics.

### Ethics considerations

Ethical clearance to conduct this study was obtained from the Medical Research Coordinating Committee (MRCC) of the Tanzania National Institute for Medical Research. Village leaders and house residents were sensitised and asked for their permission before installation of mosquito traps in their houses or premises.

## Results

### Mosquito abundance

**Fig. 3:**
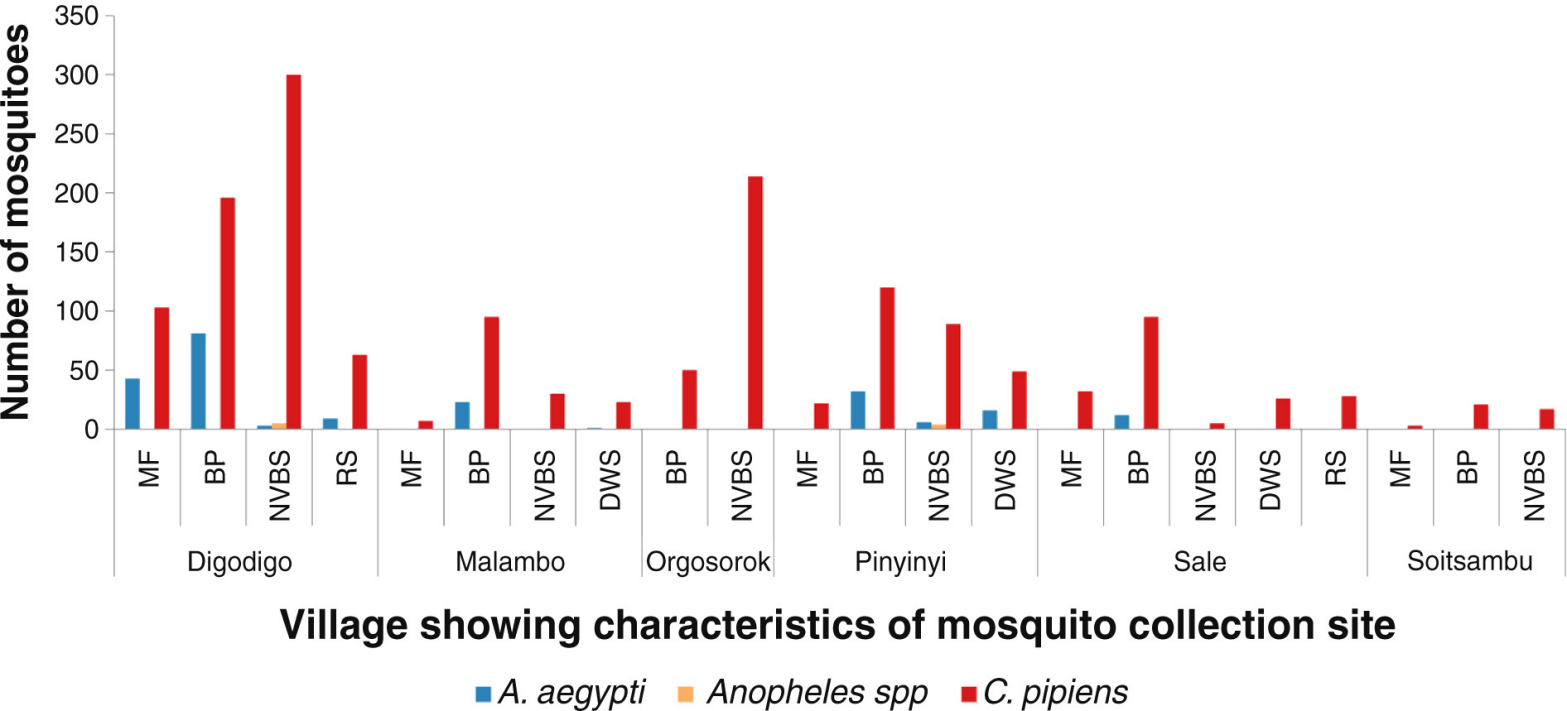
Mosquito abundance in villages according to characteristics of the mosquito collection sites. MF=maize farm; BP=banana plantation; DWS=drinking water source; NVBS=no visible breeding site; RS=river site.

**Fig. 4:**
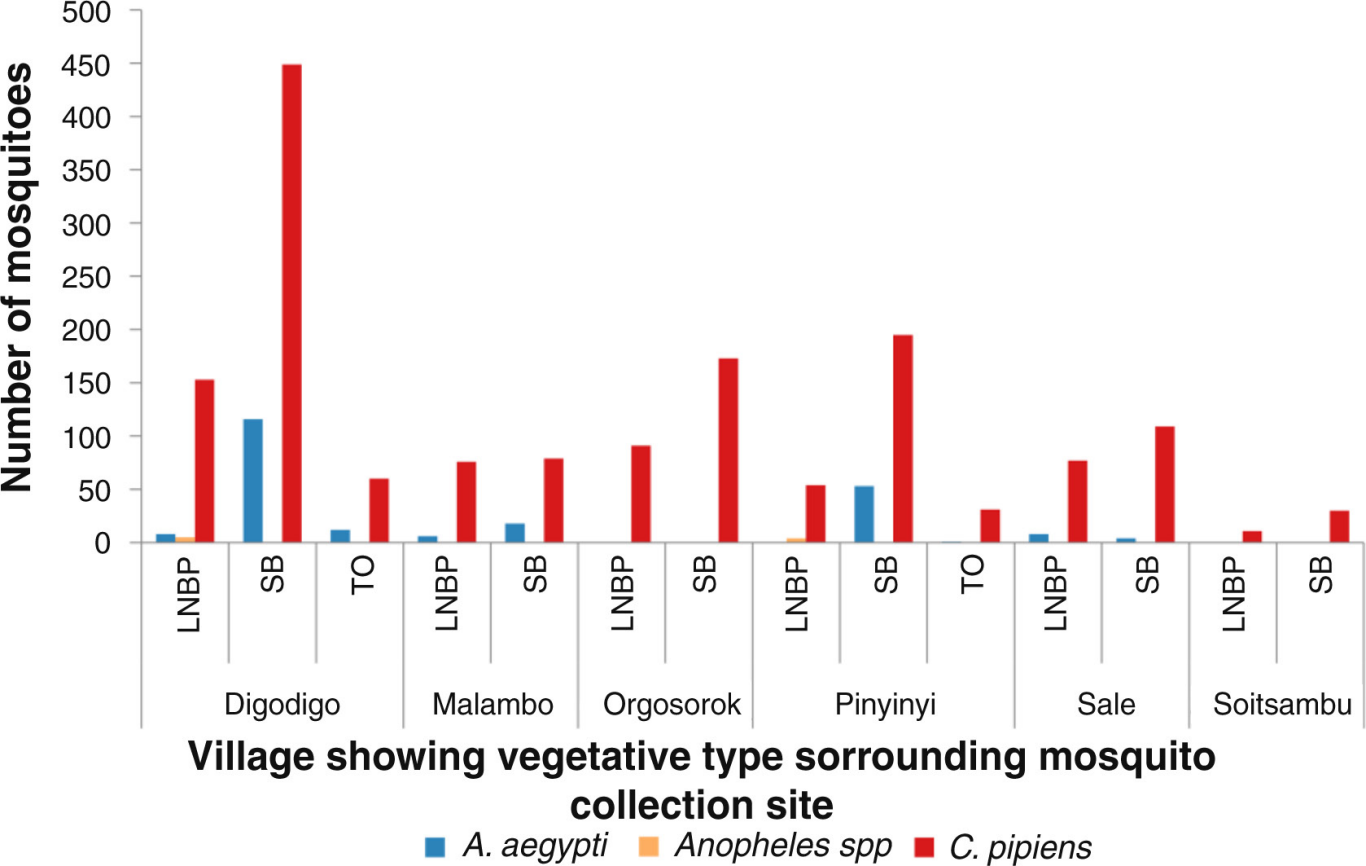
Mosquito abundance in villages according to vegetation type in proximity with mosquito sampling sites. LNBP =little or no present; SB=shrubs/bushes; TO=trees overhanging.

**Fig. 5:**
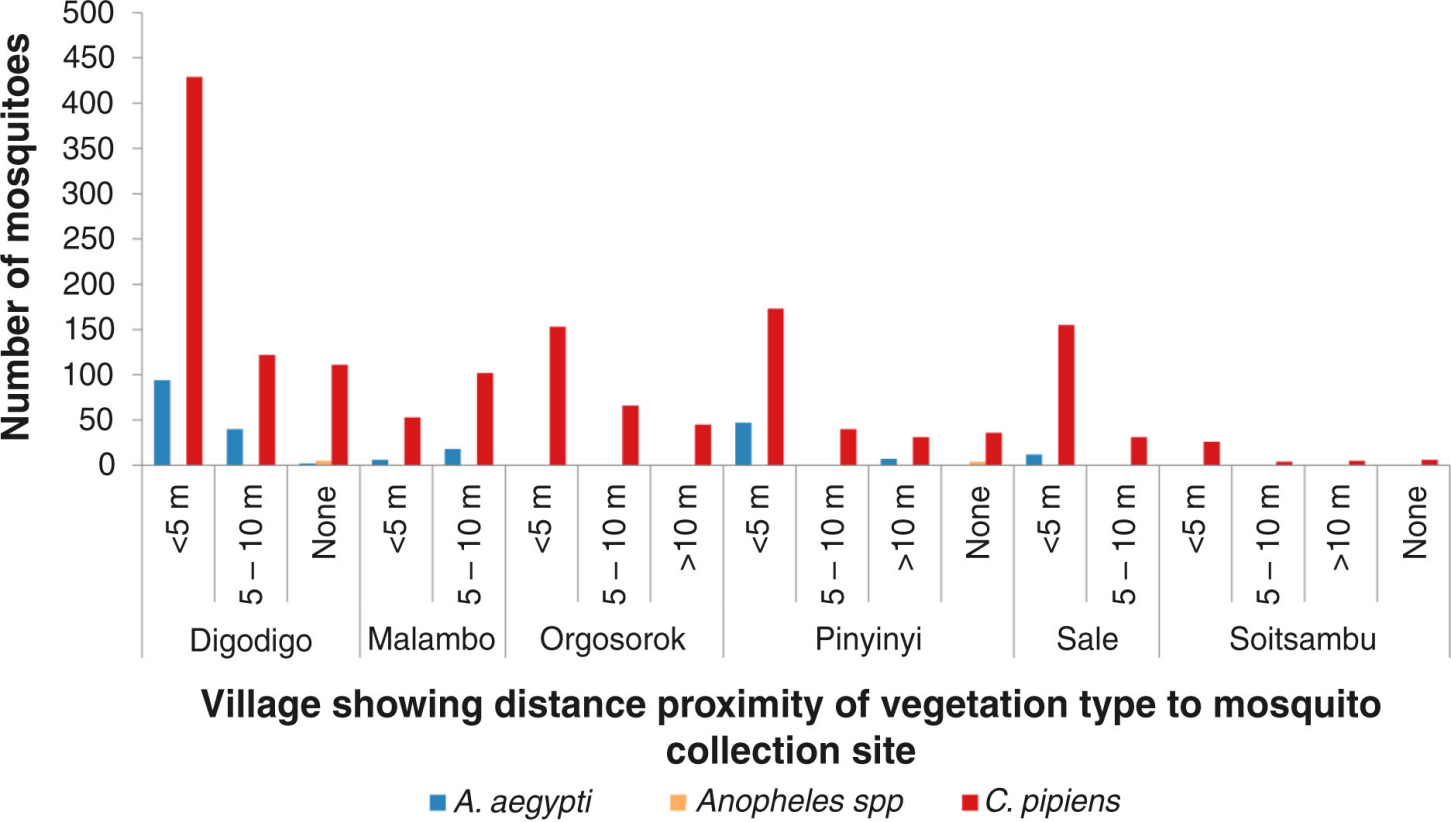
Mosquito abundance in villages according to proximity distance of the mosquito collection site to vegetation type.

**Fig. 6:**
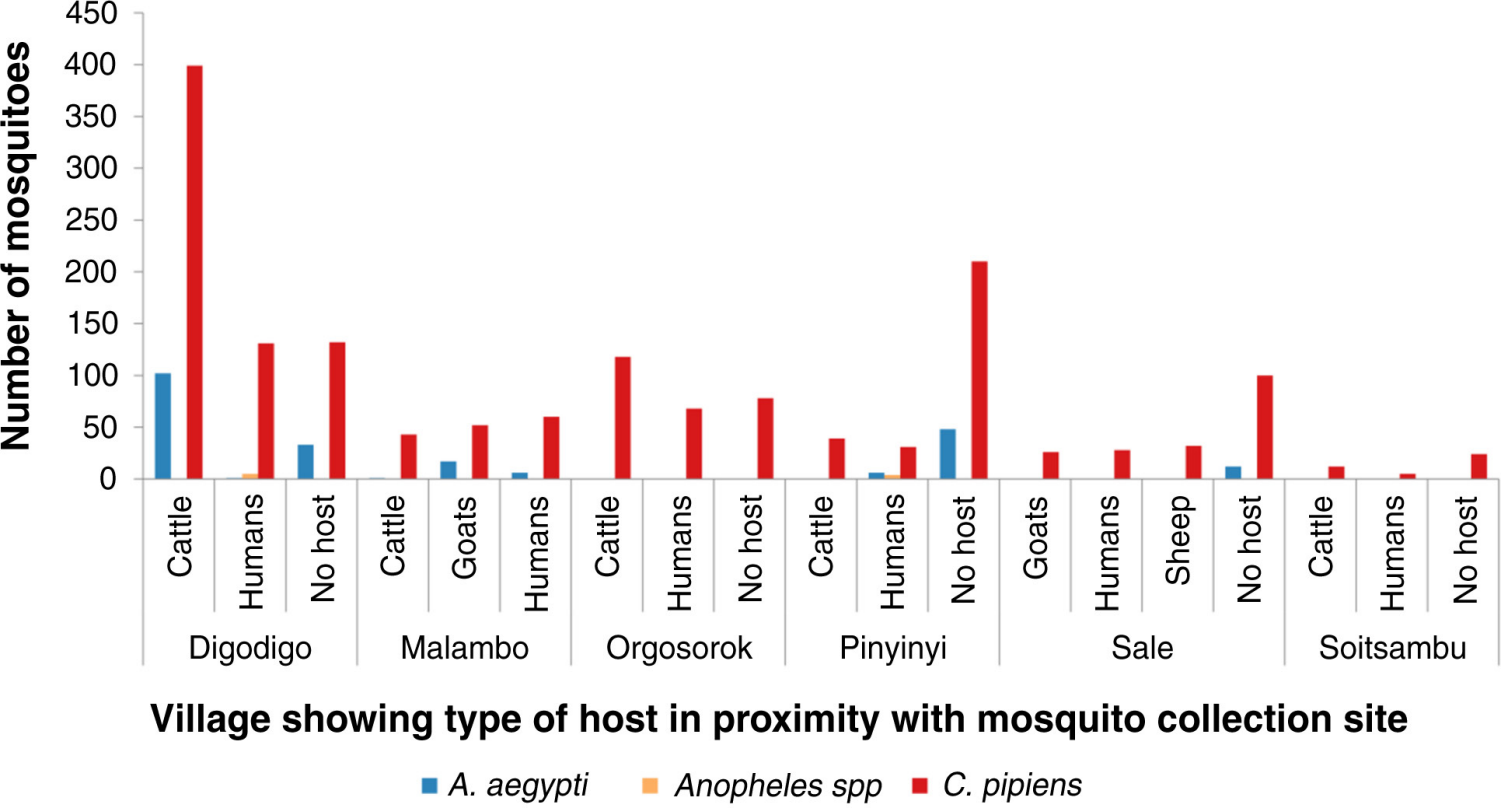
Mosquito abundance in villages according to the type of host in proximity with the mosquito collection sites.

A total of 1,823 mosquitoes were collected, of these 87% (*N*=1,588) were *C. pipiens* complex, 12% (*N*=226) *A. aegypti*, and 0.5% (*N*=9) *Anopheles* species. About two-thirds (67%; *N*=1,095) of *C. pipiens* complex and nearly 100% (*N*=225) of *A. aegypti* were trapped outdoors mainly using Mosquito Magnets. *A. aegypti* was found in four of the study sites: Digodigo (17% of 803), Malambo (13% of 179), Pinyinyi (16% of 338), and Sale (6% of 198); however, no *A. aegypti* was collected from Orgosorok and Soitsambu villages (Figs. 3–6). In Digodigo village, *C. pipiens* complex comprised 82% of the 803 total mosquitoes, Malambo 87% of 179, Orgosorok 100% of 264, Pinyinyi 83% of 338, Sale 94% of 198, and in Soitsambu 100% of the 41. All *Anopheles* species were trapped indoor using CDC light traps in Digodigo (0.6%; *N*=5) and Pinyinyi (1%; *N*=4), respectively. No *Anopheles* species were found in Malambo, Orgosorok, Sale, or Soitsambu. Of the two trapping methods used in this study, the use of Mosquito Magnets (either unbaited or baited with Octenol) captured the largest proportion of *C. pipiens* complex and *A. aegypti*. Although CDC light traps set indoor showed a low efficiency in catching *A. aegypti*, it was more efficient in capturing *C. pipiens* complex.

### Mosquito distribution

Variations in abundance of *C. pipiens* complex and *A. aegypti* among different ecological and vegetation habitats were observed. Over three quarters (78%; *N*=1,399) of *C. pipiens* complex were trapped in banana and maize farms and areas without visible breeding sites in all villages. Most (85%; *N*=191) of *A. aegypti* were mainly trapped in banana and maize farms in Digodigo, Malambo, and Sale villages. About two-thirds (65%; *N*=1,035) of *C. pipiens* complex and 70% (*N*=159) of *A. aegypti* were trapped in areas with shrubs and bushes, regardless of the proximity of livestock within the areas (Figs. 3–5). *Anopheles* species were trapped in areas without visible breeding sites or without clear vegetation type but indoor in proximity to human hosts.

Large numbers of *A. aegypti* were collected in semi-arid thorn bushes and lower Afro-montane forests of Digodigo, Malambo, and Pinyinyi villages than in arid thorn bushes areas. These three villages were the most affected areas during the 2006–2007 RVF outbreak in Ngorongoro district (Figs. 1 and 2). No statistically significant difference in abundance (*p*>0.05) was found between types of vegetation for *C. pipiens* complex.

### RVF virus detection

We tested a total of 150 pools of *C. pipiens* complex and 45 pools of *A. aegypti* organised by village and site of collection. No RVF virus was detected in any of the pools.

## Discussion

RVF epidemics have been correlated with complex processes including roles of known and unknown mosquito vectors, habitat characteristics, ecological and topographical features of vector communities, and seasonal- or climate-related vector abundances (8, 15, 29, 30, 39). In this study, the relationship between abundance of potential mosquito vectors across habitat and vegetation types experiencing different levels of RVF epidemic in Ngorongoro district was examined. Most of the recent field-based studies on RVF in Tanzania during IEP focussed mainly on the role of virus antibodies within humans, livestock, and wildlife populations (5, 40–44) . Findings of the current study show that close examination of areas favouring the emergence of massive mosquito abundance can significantly influence the emergence of disease epidemics.

Banana plantation habitats located close to human premises or animal sleeping areas were the most suitable areas for effective outdoor sampling of large numbers of mosquitoes. This distribution pattern indicates some habitat characteristic implications related to the epidemiological impact because they express the proximity of these species to humans and animals in order to effectively transmit the virus. These findings provide an important step in identifying potential areas which might influence host–vector interactions and ultimately the emergence of RVF epidemics. As data for this study were conducted during the dry season, these areas are likely to be the areas with favourable conditions for the breeding of mosquitoes especially *A. aegypti*. However, other studies (45) have reported that *A. aegypti* does not exclusively depend on the breeding grounds that emerge during the rainy period, thereby enabling this mosquito to maintain its life cycle during the dry period. Results from this study confirm findings from our previous study in the area (46) and other studies in Brazil and Italy (39, 47).

Findings from this study show that there are associations between villages that experienced previous RVF epidemics with abundance and distribution of potential mosquito vectors. Malambo and Pinyinyi villages were the most hit by the 2006–2007 RVF epidemics followed by Digodigo (5, 42). Based on our field-based observations, these villages have shown characteristic vegetation types that could predispose environmental conditions favouring major RVF epidemics and as a source of spread of infection to different areas (48). This abundance–distribution phenomenon could also explain that the disease does not spread like other classical contagious diseases but with some spreading mechanism involving animal movements locally or from neighbouring endemic countries. Since the first virus description in sheep in 1930 (1), RVF epidemics have been occurring in geographically limited areas with characteristic features and vegetation types (46). This study emphasizes further efforts to consider local-based studies of the role of mosquito abundances as a risk for disease epidemics.

In this study, we were unable to demonstrate virus activity within mosquitoes collected despite sampling being done immediately before the short rainy season. Sampling during this dry season led to a relatively small number of mosquitoes collected compared to the rainy season. Detection of RVF virus activity in mosquitoes and animals is a challenge as it requires the presence of large numbers of vectors, and the potential for this to occur is clearly limited (11, 18). It has been difficult to detect a virus in mosquitoes during IEP in Tanzania despite detection of a virus antibody among livestock and wildlife populations (4). Interestingly, it has been possible to detect the virus during IEP in *A. mcintoshi* after artificial flooding in Kenya (18). Despite unsuccessful detection of a virus, this attempt to examine virus infectivity with mosquito vectors could still add value to understanding the role of mosquitoes in the maintenance and persistence of the disease during IEP in relation to suitable sites.

Studies on vectors of RVF virus during IEP face many challenges and limitations. Lack of persistent water flooded areas during dry seasons may be an important factor in limiting the number and composition of potential RVF vectors. Absence of virus activity in this period is one of the limitations to conduct studies. Our sampling approach mainly focussed on potential vectors for RVF and on specific habitats such as banana plantations. Literature reviews suggest that attempted flooding can reveal more diversity of mosquito species relative to their abundance in disease epidemics hotspots such as Ngorongoro district (11, 18).

## Conclusions

To our knowledge, results presented here provide insights into unique habitat characterisation relating vector abundances and distribution in Ngorongoro district of northern Tanzania. Future studies should investigate long-term seasonal vector abundance in other locally known disease epidemic sites by taking into consideration the structure of the ecology and habitat distribution. Such studies could add value to strategic surveillance and control of Rift Valley fever in endemic areas.
